# Hydroethanolic Extract of Grape Peel from *Vitis labrusca* Winemaking Waste: Antinociceptive and Anti-Inflammatory Activities

**DOI:** 10.17113/ftb.60.01.22.7080

**Published:** 2022-03

**Authors:** Cristiana F. G. Silva, Victor Fattori, Caroline R. Tonetti, Marcos A. S. Ribeiro, Ricardo L. N. Matos, Jéssica B. Carra, Eduardo C. Meurer, Elisa Y. Hirooka, Janice A. Rafael, Sandra R. Georgetti, Marcela M. Baracat, Waldiceu A. Verri, Nilton S. Arakawa

**Affiliations:** 1 Department of Chemistry, State University of Londrina, Rod. Celso Garcia Cid, Km 380, Campus Universitário, 86057-970 Londrina, PR, Brazil; 2 Department of Pathology, State University of Londrina, Rod. Celso Garcia Cid, Km 380, Campus Universitário, 86057-970 Londrina, PR, Brazil; 3 Department of Exact Sciences, Federal University of Paraná, R. Dr. João Maxímiano 426, Vila Operária, 86900-000 Jandaia do Sul, PR, Brazil; 4 Department of Pharmaceutical Sciences, State University of Londrina, Av. Robert Koch 60, Operária, 86038-440 Londrina, PR, Brazil; 5 Department of Food Science and Technology, State University of Londrina, Rod. Celso Garcia Cid, Km 380, Campus Universitário, 86057-970 Londrina, PR, Brazil; 6 Pharmaceutical Science Course, Filadelfia University Center, Av. Juscelino Kubitschek 1626, Centro, 86020-000 Londrina, PR, Brazil

**Keywords:** anti-Inflammatory activity, antinociceptive effect, *Vitis labrusca* extract, winemaking waste

## Abstract

**Research background:**

Extracts from grape pomace, including the wine, show many biological effects such as antioxidant and anti-inflammatory activities. Unfortunately, winemakers discard the bagasse, so the waste is not exploited, although it contains bioactive compounds with antioxidant and anti-inflammatory properties. The work aims to analyze the hydroethanolic extract of peels from *Vitis labrusca* agro-industrial waste and to evaluate its antinociceptive and anti-inflammatory properties. This study is relevant for reusing a residue and adding value to the grape economic chain.

**Experimental approach:**

A representative sample of pomace was obtained and the peels were used to produce the extract. The phenolic compounds were determined by mass spectrometry in multiple reaction monitoring mode and Folin-Ciocalteu colorimetric method, using gallic acid as standard. The biological analyses were carried out using mice orally treated with crude extract at doses of 30, 100 and 300 mg/kg. We evaluated mechanical hyperalgesia by the von Frey method, thermal heat hyperalgesia using a hot plate at 55 °C, paw edema using a pachymeter, and neutrophil recruitment by measurement of myeloperoxidase activity. The nephrotoxicity and hepatotoxicity were evaluated by biochemical analyses using blood samples that were collected after the *Vitis labrusca* administration.

**Results and conclusions:**

In all wet winemaking residues peel mass fraction was 75%, and in dry residues 59%. We identified nine anthocyanins (3-*O*-glucosides: peonidin, delphinidin, petunidin and malvidin; 3-*p*-coumaroyl-glucosides: cyanidin, peonidin, petunidin and malvidin, and malvidin-3,5-diglucoside), five flavonoids (apigenin-7-glucoside, luteolin-7-glucoside, quercetin-3-galactoside, isorhamnetin-3-glucoside and myricetin-3-rutinoside), and mass fraction of phenolic compounds, expressed as gallic acid equivalents, was 26.62 mg/g. *In vivo* assays showed that *Vitis labrusca* extract at mass fractions 100 and 300 mg/kg reduced carrageenan-induced mechanical and thermal hyperalgesia, 50% of the paw edema, and neutrophil recruitment. In addition, there were no indications of nephrotoxicity and hepatotoxicity. Our extract obtained from winemaking residue has analgesic and anti-inflammatory properties, related at least in part to the presence of phenolic compounds, and it is not toxic to renal and hepatic tissues.

**Novelty and scientific contribution:**

This bio-product can be used as an alternative to synthetic anti-inflammatory agents with the same pharmacological potential and fewer side effects. We demonstrated that *Vitis labrusca* winemaking waste can be used for the production of antinociceptive and anti-inflammatory products (nutraceutical, pharmaceutical and cosmetics) without toxicity, contributing to the environmental economy.

## INTRODUCTION

Different communities use herbal remedies as alternative treatments for many diseases. Pain is a common symptom of health disorders and it is often a reason for seeking specialized treatment and consumption of effective drugs (*1*). Non-steroidal anti-inflammatory drugs widely used in treatment of different types of diseases cause side effects such as gastritis, ulcers, blood and renal coagulation problems, hypertension and heart failure (*2*). In this sense, in the constant search for alternative treatments, natural products represent a valuable source for discovering molecules with promising anti-inflammatory effects (*3*).

Grape pomace is composed of seeds, stems and peels, which contain a wide variety of bio-compounds, mainly phenolics. For this reason, the use of the extract obtained from grape pomace is promoted because of its various biological activities, such as antioxidant and antimicrobial activities, ability to inhibit nitrosation, and ability to modulate the activity of some enzymes (*4*-*6*). Grape contains high amounts of bioactive compounds in the form of primary and secondary metabolites with bioactive functions, mainly anti-inflammatory and analgesic activities. Chung *et al*. (*7*) verified that the phenolic compounds present in the grape can act directly on the transcription factors involved in the inflammatory response, inhibiting the expression of cytokines. Ingestion of wine and grape juice is related to the reduction of the levels of inflammatory markers such as cytokines and chemokines (*8*).

Wine has been used in diets due to synergism between polyphenols and ethanol that could be effective against chronic cardiovascular diseases (*9*). Furthermore, red wine is considered a functional food which has health-promoting properties such as antimicrobial, anti-inflammatory, anti-carcinogenic and potent antioxidant activities (*10*). At the same time, the wine industry is one of the largest producers of agro-industrial waste, and about 30% of the total volume of grapes used in industrial production becomes waste or biomass. The reuse of this residue would reduce environmental impact, such as surface and groundwater pollution, oxygen depletion, odor generation and attraction of disease-transmitting insects (*11*, *12*). Currently, in order to reduce these problems, the residue from wine production is used on a small scale as a fertilizer, a component of animal feed or in the production of distillates (*13*). Besides being a bio-product with evidence of pharmacological potential, winemaking waste can be used as an economical alternative to synthetic anti-inflammatory agents with the same pharmacological potential and fewer side effects, mainly for the pharmaceutical and cosmetic industries, making it a promising source of substances with biological activity (*14*, *15*).

Considering the pharmacological properties of phenolic (flavonoids and anthocyanins) and bioactive compounds of agro-industrial wine residues, the present study aims to identify the chemical composition of hydroethanolic extract (1:1 *V*/*V*) of *Vitis labrusca* peel. Specifically, we demonstrated *in vivo* analgesic and anti-inflammatory effects of the extract and evaluated its nephrotoxicity and hepatotoxicity.

## MATERIALS AND METHODS

### Plant material and crude extract preparation

The agro-industrial residue of *Vitis labrusca* 2014/2015 crop was supplied by Cooperativa Agro-industrial dos Viticultores (COAVITI), located in Marialva, Paraná, Brazil (23°28'20.1"S 51°48'57.9"W). The plant material consisted of peels, stems and seeds kept frozen  (-4 °C) until the preparation of the extracts. This work is duly registered with the National System of Genetic Heritage Management and Associated Traditional Knowledge (SisGen Code: A14A92F).

The grape pomace was spread over the surface of a workbench and divided in four parts. The percentage of the residue (peels, seeds and stems) from the winemaking waste was then determined by taking a quarter of the representative sample, and weighing a wet and dry mass. The whole procedure was carried out in triplicate according to the Brazilian pharmacopoeia (*16*).

The wet peels (50.0 g) were submitted to turbo-extraction (LQ 001; Metalúrgica Vithory, Catanduva, SP, Brazil) for 1 min with 250 mL of acidified hydroethanolic solution (1:1 *V*/*V*) (0.1% HCl; Synth, Diadema, SP, Brazil) followed by ultrasonication (Q3.8/40; Ultronique, Indaiatuba, SP, Brazil) at controlled temperature (max 25 °C) and protected from light for 1 h. The extract was then filtered and the extraction was repeated. The solution was removed by rotary evaporator (RC1022; Thermo Fisher Scientific, Waltham, MA, USA) at 45 °C and the concentrated extract was maintained in the freezer (-4 °C), protected from light until use.

### Chemical identification

The chemical compounds present in the extract of *Vitis labrusca* were identified using low-resolution PREMIER XE Quattro micro™ API triple quadrupole mass spectrometer with electrospray ionization (ESI-MS/MS) (Waters®, Milford, MA, USA) controlled by MassLynx v. 4.1 software. The concentration of the extracts had to be normalized for the injection of the samples and then diluted in methanol. The mass of 4.5 mg of the dried *Vitis labrusca* extract was dissolved in 1 mL methanol (UV/HPLC grade ≥99.9%; Vetec, Burlington, MA, USA), sonicated for 10 min (Ultra Cleaner 1400; Unique, Indaiatuba, SP, Brazil), centrifuged (Universal 320 R; Hettich Zentrifugen, Tuttlingen, Germany) at 4668×*g* for 10 min, and filtered through a 0.22 μm microfilter (polytetrafluoroethylene (PTFE), Thermo Fisher Scientific). Then, the sample was injected by direct infusion into the mass spectrometer by HPLC pump running in isocratic mode at a constant flow rate of 200 μL/min. All the extracts were diluted in 0.1% trifluoroacetic acid and 0.1% ammonium hydroxide solution (UV/HPLC grade ≥99.9%; Sigma-Aldrich, Merck, St. Louis, MO, USA), for the positive and negative modes respectively, in the ESI-MS/MS equipment.

The analysis parameters for multiple reaction monitoring of positive mode ionization were: argon gas flow 0.14 mL/min, desolvation temperature 250 °C, capillary voltage 3500 V, cone voltage 30 V, collision energy 30 V, syringe flow rate 50 μL/min, cycle time 2 s, and number of channels 31. For the negative ionization mode, the parameters were: argon gas flow 0.14 mL/min, desolvation temperature 250 °C, capillary voltage -2500 V, cone voltage 40 V, collision energy 30 V, syringe flow rate 50 μL/min, cycle time 2.7 s, and number of channels 27.

For the positive mode fragmentation conditions, for delphinidin-3-glucoside, ESI-MS/MS parameters were: argon gas flow 0.14 mL/min, desolvation temperature 250 °C, capillary voltage 3500 V, cone voltage 30 V, collision energy 15 V, resolution 12, and syringe flow rate 50 μL/min. For petunidin-3-glucoside and malvidin-3-glucoside they were: argon gas flow 0.14 mL/min, desolvation temperature 300 °C, capillary voltage 3500 V, cone voltage 30 V, collision energy 30 V, resolution 13, and syringe flow rate 50 μL/min.

### Total phenolic compounds

The content of total polyphenols in the extract was determined by the colorimetric method using Folin-Ciocalteu reagent (Sigma-Aldrich, Merck) and gallic acid (Vetec) as standard (*17*). A solution of extract of 2.42 mg/L was prepared and an aliquot at 0.5 mL increments were subsequently mixed with 0.5 mL Folin-Ciocalteu reagent and 0.5 mL Na_2_CO_3_ 10% (*m*/*V*) (Anidrol, Diadema, SP, Brazil). The reaction was incubated for 1 h at room temperature, and the absorbance was measured at 760 nm (Lambda 25 spectrometer; Perkin Elmer, Waltham, MA, USA). A blank sample without the extract was run under the same conditions. The calibration straight line was constructed with different concentrations of gallic acid (4-32 µg/mL) using the following equation:

y=0.03419x-0.03030 /1/

where y is the absorbance, 0.03419 is the slope, x is the gallic acid concentration (µg/mL), and 0.03030 is the linear coefficient. The absorption of the extract solution was measured according to the same procedure and the total phenolic compounds were calculated using Eq. 1 and expressed in mg gallic acid equivalents per g extract.

### Biological experimental protocol

The experiments were carried out using male Swiss mice (20-25 g) that were kept in the bioterium of the State University of Londrina, Brazil, with free access to water and feed, for two days before the experiments, using light/dark cycle 12/12 h. The animals were divided (max 12 animals per cage) into standard polypropylene cages (41 cm×34 cm×16 cm; Beira Mar Indústria e Comércio Gaiola BHG, São Paulo, SP, Brazil). They adapted to the experimental environments and conditions for at least 1 h before the experiments. The procedures for the care and handling of animals were followed according to the guidelines of the International Association for the Study of Pain (IASP; Washington, DC, USA), which was submitted to the Ethics Committee of the State University of Londrina, Brazil, for approval (protocol number 7534.2016.83).

The animals were treated orally by gavage with crude extract (peels of the grape *Vitis labrusca* and 20% Tween 80 in saline) at doses of 30, 100 and 300 mg/kg 60 min before the intraplantar stimulus with carrageenan (100 μg/animal, i.pl.).

### Evaluation of edema

Measurement of the paw volume of the animals was evaluated using a pachymeter (Tramontina PRO 300 mm, 12''; Canoas, RS, Brazil) before the inflammatory (basal) stimulus with carrageenan (100 μg/animal i.pl.) at the intervals of 1, 3 and 5 h. The results were calculated by the difference between the mean value of two measurements after the stimulus and the mean value of two measurements before the stimulus (basal) (*18*).

### Evaluation of mechanical and thermal hyperalgesia

Mechanical hyperalgesia in mice was evaluated by the von Frey method, as previously described by Cunha *et al*. (*19*). The mice were accommodated in acrylic boxes with a metal grid floor, in a quiet temperature-controlled room ((23±1) °C), 30 min before the test started. The animals were tested before (baseline) and at intervals of 1, 3 and 5 h after carrageenan stimulation (100 μg/animal i.pl.). The animals were tested by increasing a point pressure on the paw with an electronic analgesimeter (EFF 301; Insight, Ribeirão Preto, SP, Brazil), which records in N the pressure required for the animal to remove the paw. The results were calculated by the difference between the mean value of three measurements after the stimulus (1-5 h) and before the stimulus (baseline).

Thermal heat hyperalgesia was performed using a hot plate (43/03; Lucadema, São José do Rio Preto, SP, Brazil) at (55±1) °C (*20*). The animals were tested at the same intervals of 1, 3 and 5 h after the carrageenan challenge, and the results were presented as residence values (in seconds) on the hot plate. The maximum time to remain on the hot plate was 20 s to avoid tissue damage.

### Measurement of myeloperoxidase enzyme activity

Subcutaneous plantar tissue samples were collected 5 h after carrageenan stimulation (100 μg/animal i.pl.) and stored in dipotassium hydrogen phosphate buffer (pH=6.0; Anidrol) containing 0.5% hexadecyltrimethylammonium bromide. The samples were homogenized with Polytron^®^ PT 3100 D (Kinematica, Malters, Switzerland) and centrifuged (16 100×*g*, 4 °C, 2 min; Rotina 46 R; Hettich Zentrifugen, Tuttlingen, Germany) and the supernatant was collected. Aliquots of 10 μL of the supernatant sample were mixed with 200 μL of 50 mmol/L phosphate buffer solution at pH=6.0 containing 0.167 mg/mL *o*-dianisidine dihydrochloride and 0.015% hydrogen peroxide. The absorbances of the samples were recorded in a microplate spectrophotometer (Multiskan GO; Thermo Fisher Scientific) at a wavelength of 450 nm, myeloperoxidase (MPO) enzyme activity was compared to a standard neutrophil curve, and the result was expressed as myeloperoxidase activity (*21*) calculated as follows:

Enzyme activity=(*N*(neutrophils)·10^4^)/*m*(tissue) /2/

where *m* is the mass of tissue in mg.

### Renal function tests and enzymatic markers of liver injury

Blood samples were collected 5 h after the *Vitis labrusca* administration by cardiac puncture and added into microtubes containing an anticoagulant (EDTA, 5000 IU/mL; Sigma-Aldrich, Merck). The plasma was separated by centrifugation (200×*g*, 10 min, 4 °C; Rotina 46 R; Hettich Zentrifugen) and then processed according to the manufacturer's instructions (Labtest Diagnóstica S.A., Lagoa Santa, MG, Brazil) to evaluate urea and creatinine levels as indicators of nephrotoxicity, and alanine aminotransferase (ALT) and aspartate aminotransferase (AST) levels as indicators of hepatotoxicity. For renal function tests, the results were expressed in mg/100 mL of plasma urea or creatinine, and for enzymatic markers of liver injury in U/L of plasma ALT or AST (adapted from Staurengo-Ferrari *et al*. (*22*)).

### Statistical analysis

The results of the hyperalgesic parameters and all other tests were presented as mean value±SEM (standard error of the mean) of measurements performed on six animals per group with two replicates, and two-way repeated-measures ANOVA followed by the Tukey’s *post-hoc* test were used to compare the groups, and doses at all times (curves) were determined by RStudio software v. 3.4.1-2017 (*23*). For both analyses significant differences were considered for p<0.05 (adapted from Staurengo-Ferrari *et al*. (*22*)).

## RESULTS AND DISCUSSION

### Chemical analysis and phenolic compounds

The winemaking residue was composed of 75, 23 and 2% on wet mass basis, and 59, 38 and 3% on dry mass basis of peels, seeds and stems, respectively. The mass fractions on a dry mass basis of *Vitis* spp. waste are variable according to the wine production and the type of grape pomace; in the literature we found values of 51, 47 and 2% of peels, seeds and stems respectively (*24*), and 38-52% of seeds and 5-10% of peels (*25*). These mass fractions show that our work reuses most of this waste, thus enables the increase of the income of cooperatives and industries and reduces the environmental impact. In addition, even though the seeds are not evaluated in this study, they have important biological activities such as antimicrobial and antioxidant (*26*, *27*). Furthermore, the main compounds found in the grape pomace residue were unsaturated fatty acids, simple phenolics and polyphenols (*11*).

Grape pomace is rich in phenolic compounds such as anthocyanins, flavonols, flavan-3-ols, cinnamic, benzoic and ellagic acids, and stilbenes (*28*, *29*). Only the crude extract from the peels of *V. labrusca* obtained from the agro-industrial waste showed the presence of 14 compounds: apigenin-7-glucoside (*m*/*z*=431),luteolin-7-glucoside (*m*/*z*=447), quercetin-3-galactoside (*m*/*z*=463), isorhamnetin-3-glucoside (*m*/*z*=477) and myricetin-3-rutinoside (*m*/*z*=627) for negative mode; and peonidin-3-glucoside (*m*/*z*=463), delphinidin-3-glucoside (*m*/*z*=465), petunidin-3-glucoside (*m*/*z*=479), malvidin-3-glucoside (*m*/*z*=493), cyanidin-3-*p*-coumaroyl-glucoside (*m*/*z*=595), peonidin-3-*p*-coumaroyl-glucoside (*m*/*z*=609), petunidin-3-*p*-coumaroyl-glucoside (*m*/*z*=625), malvidin-3-*p*-coumaroyl-glucoside (*m*/*z*=639) and malvidin-3,5-diglucoside (*m*/*z*=655) for positive mode by ESI-MS/MS using the multiple reaction monitoring (data not shown).

The total phenolic compound mass fraction, expressed as GAE, detected *in vitro* in crude extract of *Vitis labrusca* peels was 26.62 mg/g (data not shown). Melo *et al*. (*15*) studied the extraction of Isabel grape pomace with 80% ethanol and found 16.57 mg/g of phenolic compounds. Rockenbach *et al*. (*30*) quantified the content of phenolic compounds in grape pomace (*Vitis labrusca* and *Vitis vinifera*) and obtained values between 32.62 and 74.74 mg/g. Furthermore, our study indicated that the phenolic compounds are maintained in the peels from winemaking waste. However, the types and quantity of these compounds depend on the climatic and processing conditions and the extraction method.

### Potential anti-inflammatory and analgesic effects in vivo

The reduction of paw edema, analgesic and anti-inflammatory effects were observed in our results, owing to the presence of anthocyanins, flavonols and phenolic compounds in the extract, which worked in synergism, reducing the inflammation.

**Fig. 1:**
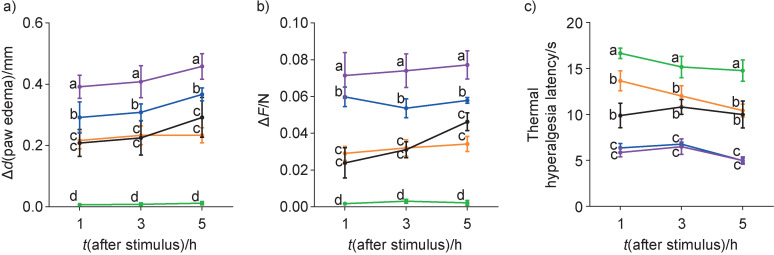
Evaluation of: a) paw edema, b) mechanical hyperalgesia as the force change, and c) thermal hyperalgesia latency 1, 3 and 5 hours after the intraplantar injection of carrageenan. The animals (*N*=6 per experimental group) were pretreated (1 h) with saline (control group, marked green), vehicle control (purple), and three doses (30 (blue), 100 (black) and 300 (orange) mg/kg) of hydroethanolic extract of the *Vitis labrusca* peels obtained from winemakers' waste. Results were expressed as mean value±standard error of the mean (different letters indicate statistical difference, p<0.05)

The extracts at the doses of 100 and 300 mg/kg reduced paw edema at all-time points when compared to the vehicle-treated group (Fig. 1a). These doses reduced 50% of the paw edema when compared to the control group after 1 and 3 h. The same trend of reduction of paw edema was presented by Figueira *et al*. (*31*) with blueberry extract, and ear edema after topical application of malvidin-3-glucoside, malvidin-3,5-diglucoside and quercetin (*32*). In contrast, the control group indicated paw edema of approx. 0.4 mm in diameter and the 30 mg/kg dose did not show statistical significance at all times (1, 3 and 5 h) for the control group.

Mechanical and thermal hyperalgesia (Figs. 1b and 1c) using a dose of 30 mg/kg of grape peel extract when compared to the carrageenan control group was not statistically different. Despite this, the extract doses of 100 and 300 mg/kg were statistically different. A sour cherry extract (400 mg/kg) containing anthocyanins showed the same effect on mechanical and thermal hyperalgesia as the indomethacin (*33*). However, these effects were seen in our study at lower doses. We tested mechanical and thermal hyperalgesia using the same model as Tall *et al*. (*33*); unfortunately, their study did not characterize the extract, and biological results depend on the types and amount of compounds present in the extracts.

The inflammatory pain results from increased sensitization of peripheral nociceptors resulting from the stimulation of pro-inflammatory mediators by cytokines and chemokines. The regulation of the analgesic pathway is very complex, where the anthocyanins have anti-inflammatory and antioxidant properties, acting in many phases of the pathway (*34*). In addition, the anthocyanins might have these effects owing to the displacement of π electrons generated by the conjugation extension present in their structures. Moreover, the central ring, or ring B, acts as an electrophile species capable of undergoing nucleophilic addition (*35*), which reacts on the enzymatic pathway.

**Fig. 2:**
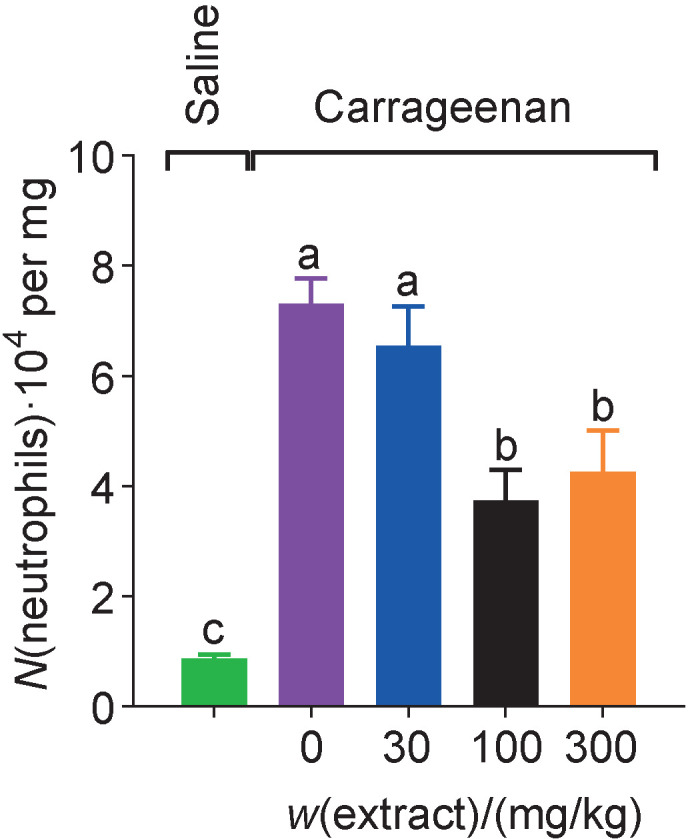
Evaluation of myeloperoxidase (MPO) activity after 5 h of intraplantar injection of carrageenan, expressed as number of neutrophils per mg of animal tissue. The mice (*N*=6 per experimental group) were treated with saline (control group, marked green), vehicle control (purple), and three doses (30 (blue), 100 (black) and 300 (orange) mg/kg) of hydroethanolic extract of *Vitis labrusca* peels obtained from winemakers' waste 1 h before the carrageenan-induced inflammation. Results were expressed as mean value±standard error of the mean (different letters indicate statistical difference, p<0.05)

One of the inflammatory triggers might be evaluated by the quantification of MPO, which is linked to the injured and reactive species of the tissues. The carrageenan stimulation significantly increased MPO activity after 5 h (Fig. 2); in contrast, 100 and 300 mg/kg of peel extract from *V. labrusca* from winemakers' waste statistically significantly reduced the MPO. Herein, the extract of *Malva sylvestris*, which contains anthocyanins, their isolates and flavonol, culminated in the same results (*35*).

**Fig. 3:**
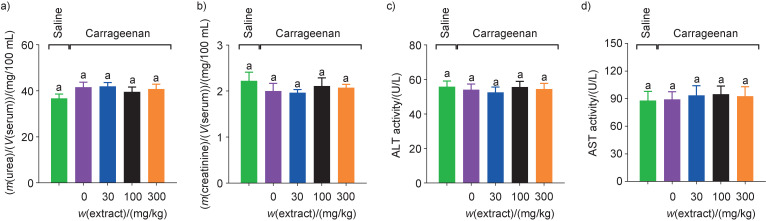
Evaluation of biochemical parameters of serum: a) urea, b) creatinine, c) alanine aminotransferase (ALT), and d) aspartate aminotransferase (AST) after 5 h of intraplantar injection of carrageenan. The mice (*N*=6 per experimental group) were treated with saline (control group, marked green), vehicle control (purple), and three doses (30 (blue), 100 (black) and 300 (orange) mg/kg) of hydroethanolic extract of *Vitis labrusca* peels obtained from winemakers' waste 1 h before the carrageenan-induced inflammation. Results were expressed as mean value±standard error of the mean (different letters indicate statistical difference, p<0.05)

Medications can be toxic, causing liver, kidney and other tissue damage. Biochemical tests are commonly used to observe changes in liver cells, monitored by enzymes such as AST and ALT, as well as blood urea and creatinine concentrations that may or may not demonstrate possible renal changes. All biochemical parameters evaluated in this study remained much the same regardless of treatment, with no significant difference (Fig. 3), similar to the observations of Figueira *et al*. (*31*). Thus, the toxicity of the tested grape skin extract was not demonstrated, since anthocyanins, flavonoids and phenolic compounds are natural metabolites suggestive of low or no toxicity in liver and kidney, when evaluated by blood biochemical parameters.

Winemaking residue can be utilized for combustion, pyrolysis (*24*), and in food, pharmaceutical and cosmetic industries as an alternative raw material (*36*), generating incomes for the wineries and minimizing environmental impact. In addition, anthocyanins are healthy and safe compounds that have anti-inflammatory and antioxidant properties (*34*); grape pomace, in addition to anthocyanins, contains flavonoids and phenolic compounds with similar properties.

## CONCLUSIONS

Our work reuses the grape peels present in the majority of winemaking residues, and the crude extract demonstrated analgesic and anti-inflammatory activities, related at least in part to the presence of phenolic compounds. We also showed that there was no toxicity to renal and hepatic tissues. Therefore, this discarded residual biomass can be considered a cheap and widely available source for the extraction of phenolic compounds, demonstrating a possible alternative to anti-inflammatory and analgesic drugs, generating economic gains and minimizing environmental impacts. In addition, the remainder of the pomace might be evaluated in future studies.
